# Determination of M1/M2 Macrophage Polarization in Ipsilateral and Contralateral Rat Testis Tissue Following Unilateral Torsion/Detorsion

**DOI:** 10.1007/s43032-024-01519-6

**Published:** 2024-03-26

**Authors:** Merve Kavak Balgetir, Nalan Kaya Tektemur, Ahmet Tektemur, Gaffari Türk, İbrahim Halil Güngör, Aslıhan Cakir Cihangiroglu, Ramazan Fazıl Akkoç, Tuncay Kuloglu, Durrin Ozlem Dabak

**Affiliations:** 1 Department of Histology and Embryology, Fethi Sekin City Hospital, Elazig, 23119 Turkey; 2 Department of Histology and Embryology, Faculty of Medicine, Firat University, Elazig, Turkey; 3 Department of Medical Biology, Faculty of Medicine, Firat University, Elazig, Turkey; 4 Department of Reproduction and Artificial İnsemination, Faculty of Veterinary Medicine, Firat University, Elazig, Turkey; 5 Department of Anatomy, Faculty of Medicine, Firat University, Elazig, Turkey

**Keywords:** Testis. Torsion, Detorsion. M1, M2 Macrophages

## Abstract

The present study investigates the changes in M1/M2 macrophage polarization resulting from unilateral testicular torsion in the bilateral testis. The study sample included 63 male Sprague–Dawley rats, which were randomly divided into nine groups (n = 7): Control, Sham (4 h (4 h), 24 h, 7 days (7d), 14d), and Torsion/Detorsion (T/D) (4 h, 24 h, 7d, 14d). Histopathological evaluations revealed no changes in the Sham groups, while T/D was noted to cause edema, vascular occlusion, disruption of seminiferous tubule epithelial organization, germ cell abnormalities and structural anomalies in the experimental rats, the severity and extent of which increased from 4 h to 14d after T/D. The Cosentino scores used to determine the degree of histological damage were consistent with the histopathological findings in all groups, while the Johnsen scores, as a marker of spermatogenesis, were lower in the T/D groups. Seminiferous tubule diameters and germinal epithelial thickness decreased significantly in parallel with increased tubule damage in the ipsilateral testicles. Testicular torsion significantly affected sperm motility, with significant reductions observed in the T/D 7d and T/D 14d groups. A hormone profile analysis revealed decreased testosterone levels in both the Sham and T/D groups when compared to the Controls. CD68 and CD163 immunoreactivities, as M1 and M2 macrophage surface markers, were determined in the testicular tissue using the avidin–biotin-peroxidase complex method. T/D interventions caused M1/M2 macrophage polarization changes and increased M1 macrophages, particularly in contralateral testicular tissue. The increase in M1 macrophages in contralateral testicular tissue following T/D in the present study suggests that cell processes, including macrophages, may play an important role in contralateral testicular injury.

## Introduction

Testicular torsion is defined as the rotation of the testis and the twisting of the spermatic cord around itself. The primary pathophysiology of testicular torsion involves the rotation of the spermatic cord, leading to ischemia (restricted blood supply) in the tissue. The subsequent untwisting of the rotated cord results in reperfusion, leading to ischemia–reperfusion injury in the testis, which has been linked to increased production of proinflammatory cytokines and adhesion molecules, neutrophil release, reactive oxygen species formation, lipid peroxidation, apoptosis, anoxia and changes in microvascular blood flow [1, 2].

After torsion of the testicle, damage occurs in the bilateral (both sides) testicles with varying degrees of severity. The possible damage to the contralateral testicles following detorsion is associated with several mechanisms, including disruption of the blood-testis barrier, autoimmune reactions against spermatogonia, sympathetic reflex responses, excessive nitric oxide (NO) production and increased production of reactive oxygen species (ROS), although the exact pathophysiology of contralateral testicular damage following testicular torsion is still not fully understood [3, 4]. Testicular loss, infertility, infection, reduced exocrine and endocrine function, and cosmetic deformity are possible complications following testicular torsion, due either to inadequate or delayed interventions or incidentally [5]. The testes possess a specific immunological environment due to their role as a privileged organ, offering local natural defense against microbial infections and the ability to prevent autoimmune responses against male germ cells [6]. Macrophages located in the interstitial area are believed to play a significant role in the maintenance of testicular immune privilege [7]. Macrophages in rat testes constitute approximately 20% of the interstitial cell population.

The immunoregulatory phenotype of testicular macrophages is essential for the maintenance of normal testis homeostasis [8]. Macrophages are cells of the mononuclear phagocytic system and are known to have important functions in both non-specific and antigen-specific immune responses, although they should not be thought of solely as immune cells [9]. Typically situated in tissues, they integrate incoming signals upon stimulation, conveying instructions to neighboring stromal cells to maintain tissue integrity and thus contribute to homeostasis [10]. Macrophages can also be defined as heterogeneous and dynamic cell populations that can polarize into different types in response to various stimuli and adapt to different environments. This polarization ability allows macrophages to adapt to changing environments and even undergo cellular changes depending on the stimuli and the state of the local microenvironment, but they may not always polarize or clonally expand into a single type under different stimuli, and their functions may change according to the cytokine profile of the microenvironment, which is related to the recognition of stimuli and receptors [11]. To date, two general macrophage polarization phenotypes have been identified: classically activated M1 macrophages, and alternatively activated M2 macrophages [12]. Classically activated M1 macrophages are proinflammatory cells that are activated by LPS, IFN and GM-CSF, and produce high levels of such cytokines as TNF-α, IL-1, IL-6, IL- 12 and IL-23 [13]. In contrast, alternatively activated M2 macrophages are involved in tissue homeostasis and immune regulation, and have anti-inflammatory effects. Macrophages polarize into the M2 phenotype through stimulation with such mediators as IL-10, TGF-β, glucocorticoids, IL-4 and IL-13 [14]. There are various pathological processes behind the changes in the polarization of testicular macrophages, which can differentiate into M1 cells via IFN-γ or into M2 cells via IL-4 in cases of unilateral testicular torsion [15, 16].

Although the mechanism has yet to be fully elucidated, testicular macrophages also play a role in tissue repair in response to inflammation [17]. Studies of testicular torsion/detorsion have reported damage in both the ipsilateral and contralateral testicles. Despite the many studies of this topic to date, the mechanism behind contralateral testicular damage is still to be fully explained. Furthermore, there have been no studies to date investigating whether changes occur in M1/M2 macrophage polarization in both the ipsilateral and contralateral testes following unilateral testicular torsion, or whether such changes play a role in the pathophysiology of testicular torsion/detorsion. The present study investigates the changes in M1/M2 macrophage polarization in both the ipsilateral and contralateral testes at different time intervals following unilateral testicular torsion.

## Materials and Method

### Experimental Animals

The study was approved by the local ethics committee of the Firat University Faculty of Medicine (Ethics no: 11.03.2020, 2020/04), and was conducted with adherence to the guidelines of the European Union (2010/63/EU). A total of 63 adult male Sprague–Dawley rats aged 8–10 weeks were acquired for the study from the Firat University Experimental Research Center (FÜDAM). All procedures related to the animals were carried out at FÜDAM following the Animal Research: Reporting of In Vivo Experiments guidelines.

### Spermatic Cord Torsion

The rats were randomly divided into nine groups, each containing seven rats, and assigned as the Control group, Torsion groups (T/D 4 h, T/D 24 h, T/D 7d and T/D 14d) and Sham groups (Sham 4 h, Sham 24 h, Sham 7d and Sham 14d). The control group underwent no treatment during the experimental period, while in the torsion groups, the tunica vaginalis was dissected and the left testis was rotated 720° clockwise around its axis along the spermatic cord. The twisted left testis was secured with a silk suture and placed back into the scrotum, which was covered with.

A sterile gauze pad for 4 h and regularly moistened with normal saline to prevent. drying. At the end of the 4th hour, detorsion was performed, the testis was returned to the scrotal sac and the skin was closed with a VICRYL suture. In the sham groups, the tunica vaginalis was dissected and the left testicle was removed and kept outside for 4 h, and underwent no procedure other than being covered with a sterile gauze and moisturized. At the end of the 4th hour, the testicle was returned to the scrotal sac and the skin was closed with VICRYL sutures. All surgical operations were performed under aseptic conditions and ketamine (50 mg/kg, intraperitoneal) anesthesia. The experimental procedures in the T/D groups were terminated at the 4th hours, 24th hours, 7th days, and 14th days following torsion, and following the surgical operation in the Sham groups. The testicles were removed from the rats after decapitation under general anesthesia (80 mg/kg ketamine and 12 mg/kg xylazine) [18], the testis tissue samples were fixed in Bouin's fixative for histological evaluation, and approximately 4–5 mL of trunk blood was collected from the decapitation site and centrifuged (3500 rpm for 5 min) to separate the serum. The serum samples were then stored at -20 °C until hormone assays were conducted.

### Histological Evaluation

The testicular tissues fixed in Bouin’s solution were processed through a routine histological follow-up series for the preparation of paraffin blocks. Sections with a thickness of 5 µm were subjected to hematoxylin and eosin (H&E), periodic acid–Schiff (PAS), and Mallory–Azan staining, followed by examination and photography under a light microscope (Leica DM 2500; Wetzlar, Germany).

Fifty randomly selected round or near-round seminiferous tubules were selected from different areas in each group, and the diameters of the seminiferous tubules and the thicknesses of the germinal epithelium were recorded [19]. The Cosentino classification was used for the assessment of the overall histopathological changes in the groups [20], while spermatogenesis was evaluated using Johnsen’s average testicular biopsy score criteria [21].

### Immunohistochemical Evaluation

The avidin–biotin-peroxidase complex method was used to determine the immunoreactivities of macrophage surface markers CD68 and CD163 (KP1 Mouse Monoclonal Antibody, sc-20060, Santa Cruz Biotechnology, Inc., California, USA; ED2 Mouse Monoclonal Antibody, sc-58965, Santa Cruz Biotechnology, Inc., California, USA, respectively) in the testis tissue and the determination of the M1/M2 macrophage polarization. The slides were counterstained with hematoxylin, and the immunohistochemical histoscores were established based on the prevalence (0.1: < 25%, 0.4: 26–50%, 0.6: 51–75%, 0.9: 76–100%) and severity (0: none, + 0.5: very little, + 1: little, + 2: medium, + 3: severe) of immunoreactivity, while the histoscore was calculated based on prevalence and severity (Histoscore = prevalence × severity).

### Sperm Analysis

The right epididymis was compressed for 2 min, allowing all spermatozoa in the epididymal tissue to pass into the liquid. The spermatozoa were counted in the diluted supernatant in both counting areas of the Neubauer chamber at × 200 magnification.

For motility evaluation, a section was prepared from the left cauda epididymis, and a suspension of 5–10 µl containing spermatozoa was prepared by homogenously mixing it with a Tris buffer solution. Three different areas were randomly examined in a single droplet of the suspension and the average values recorded for the three different areas were recorded as the motility rate percentages.

To determine the abnormal spermatozoa rate, eosin-nigrosin stain was added to the Tris buffer–spermatozoa mixture, and thin smears were prepared from this mixture and dried. The smears were examined at × 400 magnification, a total of 200 spermatozoa were examined in each smear and the rate of abnormal spermatozoa was expressed as a percentage [22].

### Hormone Assays

Testosterone (T) (SunRed; REF: SRB-T-86337 LOT:202,102), follicle-stimulating hormone (FSH) (SunRed; REF: 201,110,183 LOT:202,102) and luteinizing hormone (LH) (SunRed; REF: 201,110,180 LOT:202,102) were assessed in the rat serum samples using the ELISA (Enzyme-linked immunosorbent assay) method. The optical density of the FSH, LH and T plates was measured at 450 nm using a plate reader (Thermo Scientific), and the recorded sensitivities of the assays were 0.202 IU/L for FSH, 0.206 mIU/mL for LH and < 8.775/mL for T.

### Statistical Analysis

IBM SPSS Statistics for Windows (Version 22.0. Armonk, NY: IBM Corp.) was used for the statistical analysis of the data, presented as mean ± standard deviation (SD). For all analyses, statistical significance was considered when *p* < 0.05. The normality of the variable distribution was confirmed with a Shapiro–Wilk test, and an Analysis of Variance (ANOVA) with post hoc Tukey HSD was employed for the evaluation of the normally distributed data.

## Results

### Histopathological Effects of Testicular I/R on the Ipsilateral and Contralateral Testis

**Fig. 1:**
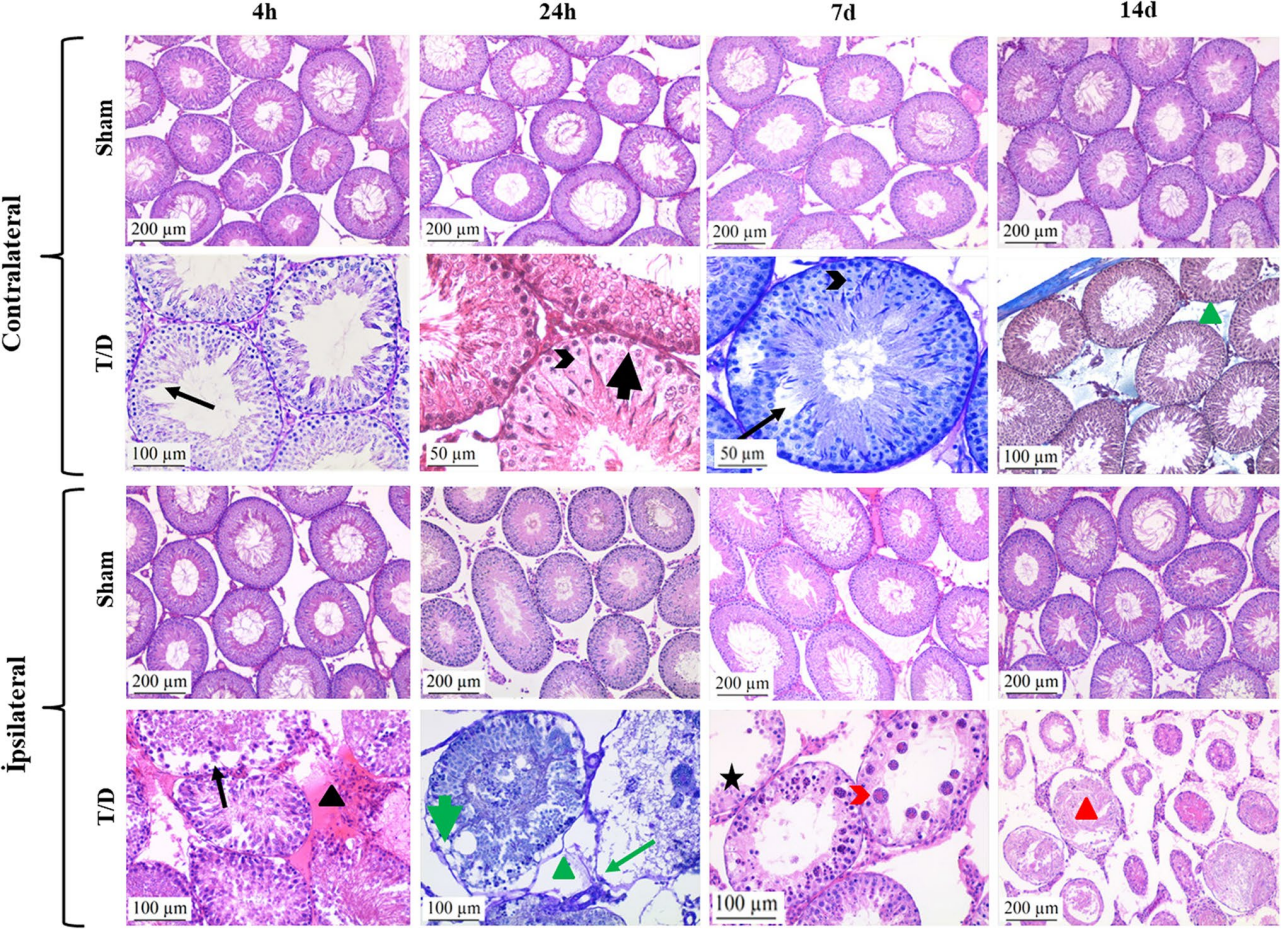
Photomicrographs of the contralateral and ipsilateral testes of rats (H&E, PAS or Azan Mallory stains). In the bilateral testes of the Sham groups, a normal appearance of seminiferous tubules was observed. Contralateral testes in the T/D 4 h group (H&E) exhibited disrupted germinal cell layers (black thin arrow), and those in the T/D 24 h group (Azan Mallory stain) exhibited vacuolization (black thick arrow) and metaphase arrest (black arrow head). Metaphase arrest (black arrow head) and disrupted germinal cell layers (black thin arrow) were noted in the T/D 7d (PAS) group, while edema (green triangle) was observed affecting the interstitial area in the T/D 14d (Azan Mallory stain) groups. An evaluation of the ipsilateral testes of the T/D 4 h group (H&E) revealed hemorrhage (triangle), the disruption of seminiferous tubule epithelial organization and degeneration (black arrow). In the T/D 24 h group (PAS), separations in the basement membrane (green thin arrow), edema (green triangle), and vacuolization (green thick arrow) were observed. In the T/D 7d group (H&E), degeneration of the seminiferous tubules (black star) and multinucleated giant cells (red arrowhead) were observed, while in the T/D 14d group (H&E), degenerated and necrotic tubules (red triangle) were detected

Testicular damage was evaluated based on H&E staining. As shown in Fig. 1, testicular T/D induced significant tissue injury, including edema, vascular congestion, seminiferous tubule degeneration, vacuolization and immature cell debris in the seminiferous tubule lumen of both the ipsilateral and contralateral testes.

Histopathological changes in the T/D 4 h group were similar in both testicles and were as mentioned above. Separations in the seminiferous tubule basement membrane and degeneration in some seminiferous tubules were observed in the ipsilateral testis tissue in the T/D 24 h group. In the T/D 7d group, seminiferous tubule degeneration and multinucleated giant cells were detected in the ipsilateral testis indicating increased histopathological changes and the onset of irreversible damage, while when the ipsilateral testicles were evaluated in the T/D 14d group, many degenerated and necrotic tubules were noted. In the contralateral testicles, the pathological findings in the T/D 4 h group were more severe in the 24 h, 7d and 14d groups, and metaphase arrest was observed in the spermatocytes in the T/D 24 h, 7d and 14d groups in the tissues on this side (Fig. 1).

### Seminiferous Tubule Diameter and Germinal Epithelium Thickness

A comparison of the contralateral testes in the Sham and T/D groups from the same time/day revealed no significant difference in seminiferous tubule diameters (STD) or germinal epithelium thicknesses (GET) (*p* > 0.05).

In the ipsilateral testes, the STDs were noted to have decreased significantly in the Sham 24 h, Sham 7d, Sham 14d, T/D 24 h, T/D 7d, and T/D 14d groups when compared to the Control group (*p* < 0.05). A comparison of only the T/D groups revealed a progressive decrease in STDs, reaching the lowest value in the T/D 14d group (*p* < 0.05). On the same side, in the T/D 7d and T/D 14d groups, the GETs were reduced more than in all other groups, to a statistically significant degree (*p* < 0.05) (Table 1).

**Table 1:** Seminiferous Tubule Diameter and Germinal Epithelium Thickness in Testicular Tissue

Ipsilateral (Left)				Contralateral (Right)	
	N	Germinal epithelial thickness	Seminiferous tubule diameter	Germinal epithelial thickness	Seminiferous tubule diameter
Control	50	86.471 ± 1.370^ h,ı^	302.974 ± 2.887	88.695 ± 1.639	311.429 ± 4.265
Sham 4h	50	75.666 ± 3.728^ h,ı^	295.199 ± 3.378^c^	80.241 ± 1.384	273.923 ± 3.335^a,d,e^
Sham 24h	50	78.473 ± 0.957^ h,ı^	262.833 ± 2.381^a,b,d,e^	79.425 ± 1.494	287.465 ± 4.026^a^
Sham 7d	50	74.713 ± 1.138^ h,ı^	282.321 ± 3.089^a,c^	81.946 ± 1.071	299.952 ± 4.350^b,e^
Sham 14d	50	78.192 ± 1.374^ h,ı^	283.985 ± 2.714^a,c^	80.022 ± 5.515	302.930 ± 3.457^b^
T/D 4h	50	77.609 ± 1.678^ h,ı^	305.972 ± 2.866^c,d,e^	78.118 ± 1.114	284.160 ± 2.985^a,e^
T/D 24h	50	79.517 ± 1.366^ h,ı^	268.470 ± 3.285^a,b,c,f^	79.243 ± 1.423	273.233 ± 4.361^a,d,e^
T/D 7d	50	63.905 ± 3.505^ı^	256.646 ± 6.273^a,b,d,e,f^	85.538 ± 1.360	295.991 ± 3.512^b,g^
T/D 14d	50	32.839 ± 1.314^ h^	198.061±3.005^a,b,c,d,e,f,g,h^	87.054 ± 1.602	305.627 ± 3.879^b,c,f,g^
*p* values		0.001	< 0.001	0.089	< 0.001

Values are presented as mean ± standard error. ^a^: Compared with the Control group; ^b^: Compared with the Sham 4 h group; ^c^: Compared with the Sham 24 h group; ^d^: Compared with the Sham 7d group; ^e^: Compared with the Sham 14d group; ^f^: Compared with the T/D 4 h group; ^g^: Compared with the T/D 24 h group; ^h^: Compared with the T/D 7d group; ^ı^: Compared with the T/D 14d group; *p* < 0.05

### Johnsen and Cosentino Scoring

Cosentino scoring, indicating the histopathological changes that occur in testicular tissue, revealed a significant increase in the ipsilateral testicular tissue of the T/D 7d and T/D 14d groups when compared to the Control group (*p* < 0.05).

In the statistical analysis of the Johnsen scoring, which is used to evaluate spermatogenesis, the scores decreased in the ipsilateral testes in the T/D 7d and T/D 14d groups compared with the other torsion groups (*p* < 0.05) (Table 2).

**Table 2:** Histopathological Scoring Table

Ipsilateral (Left)				Contralateral (Right)	
	N	Johnsen’s Score	Cosentino Score	Johnsen’s Score	Cosentno Score
Control	50	9.660 ± 0.068	1.20 ± 0.447	9.680 ± 0.067	1.20 ± 0.447
Sham 4h	50	9.280 ± 0.064^a^	1.40 ± 0.548	9.500 ± 0.071	1.40 ± 0.548
Sham 24h	50	9.260 ± 0.063^a^	1.60 ± 0.548	9.440 ± 0.071	1.60 ± 0.548
Sham 7d	50	9.300 ± 0.065^a^	1.60 ± 0.548	9.480 ± 0.071	1.20 ± 0.447
Sham 14d	50	9.300 ± 0.065^a^	1.20 ± 0.447	9.520 ± 0.071	1.00 ± 0.000
T/D 4h	50	5.720 ± 0.125^a,b,c,d,e^	2.60 ± 0.548	6.640 ± 0.098^a,b,c,d,e^	1.80 ± 0.837
T/D 24h	50	4.520±0.174^a,b,c,d,e,f^	2.80 ± 0.447	6.080 ± 0.223^a,b,c,d,e^	2.40 ± 0.548
T/D 7d	50	2.740±0.131^a,b,c,d,e,f,g^	3.60 ± 0.548^a,b,e^	4.860 ± 0.131^a,b,c,d,e^	2.60 ± 0.548
T/D 14d	50	3.180±0.093^a,b,c,d,e,f,g^	3.80 ± 0.447^a,b,c,d,e^	4.940 ± 0.105^a,b,c,d,e^	3.00 ± 0.707
*p* values		< 0.001	< 0.001	< 0.001	< 0.001

Values are presented as mean ± standard error. ^a^: Compared with the Control group; ^b^: Compared with the Sham 4 h group; ^c^: Compared with the Sham 24 h group; ^d^: Compared with the Sham 7d group; ^e^: Compared with the Sham 14d group; ^f^: Compared with the T/D 4 h group; ^g^: Compared with the T/D 24 h group; ^h^: Compared with the T/D 7d group; ^ı^: Compared with the T/D 14d group; *p* < 0.05

### Immunohistochemical Findings

**Fig. 2:**
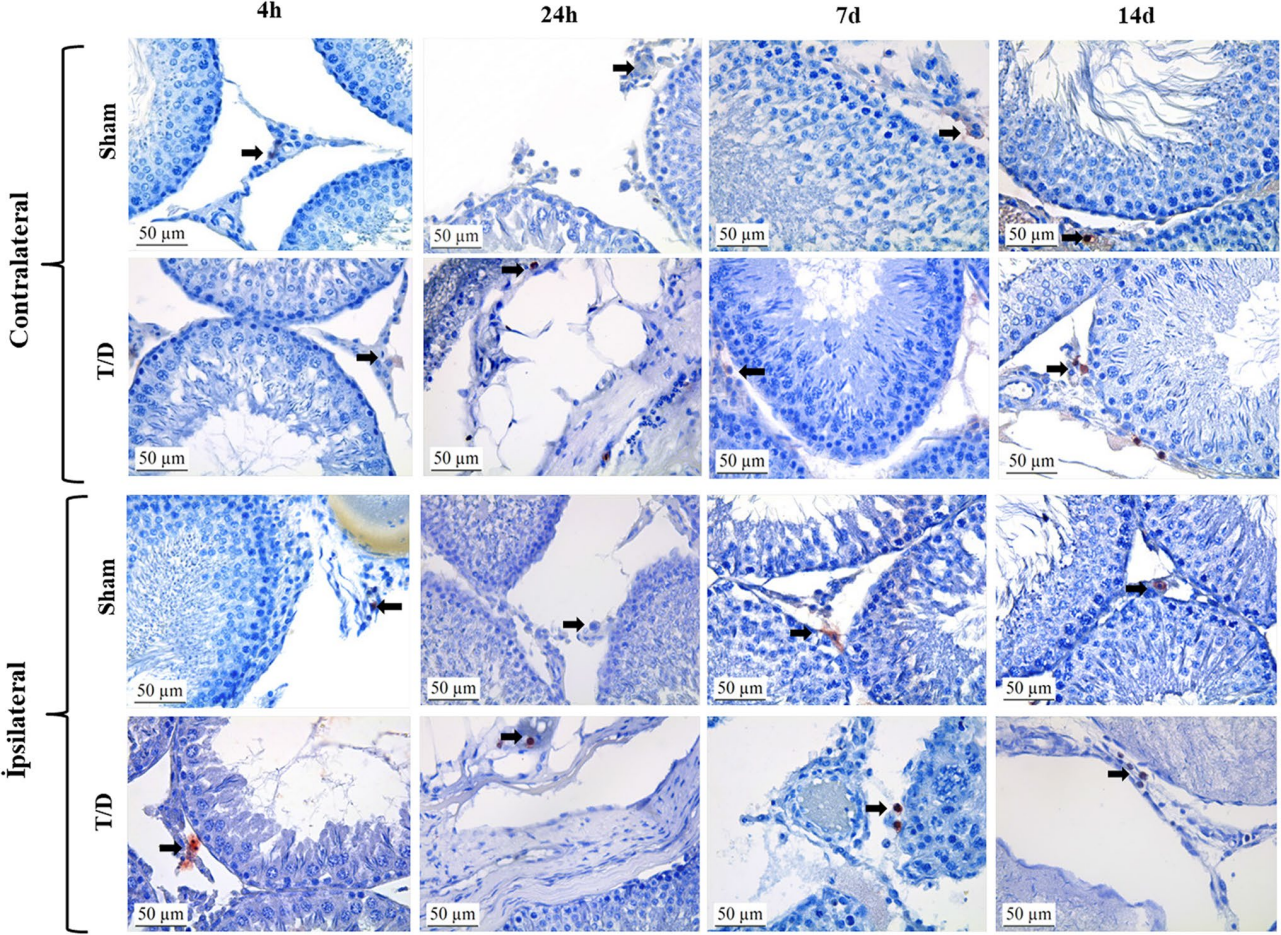
Immunohistochemical analysis of CD68 protein expression in testis tissue. Positive staining is indicated by a dark brown color (black arrows). All slides were counterstained with hematoxylin, scale bar: 50 μm, × 400

In the ipsilateral testicular tissues, the immunoreactivity of the M1 macrophage marker CD68 was noted to increase in a comparison of the T/D 24 h and T/D 14d groups. In the T/D 14d group, CD68 immunoreactivity differed significantly from that of the Control group (*p* < 0.05) (Fig. 2).

**Fig. 3:**
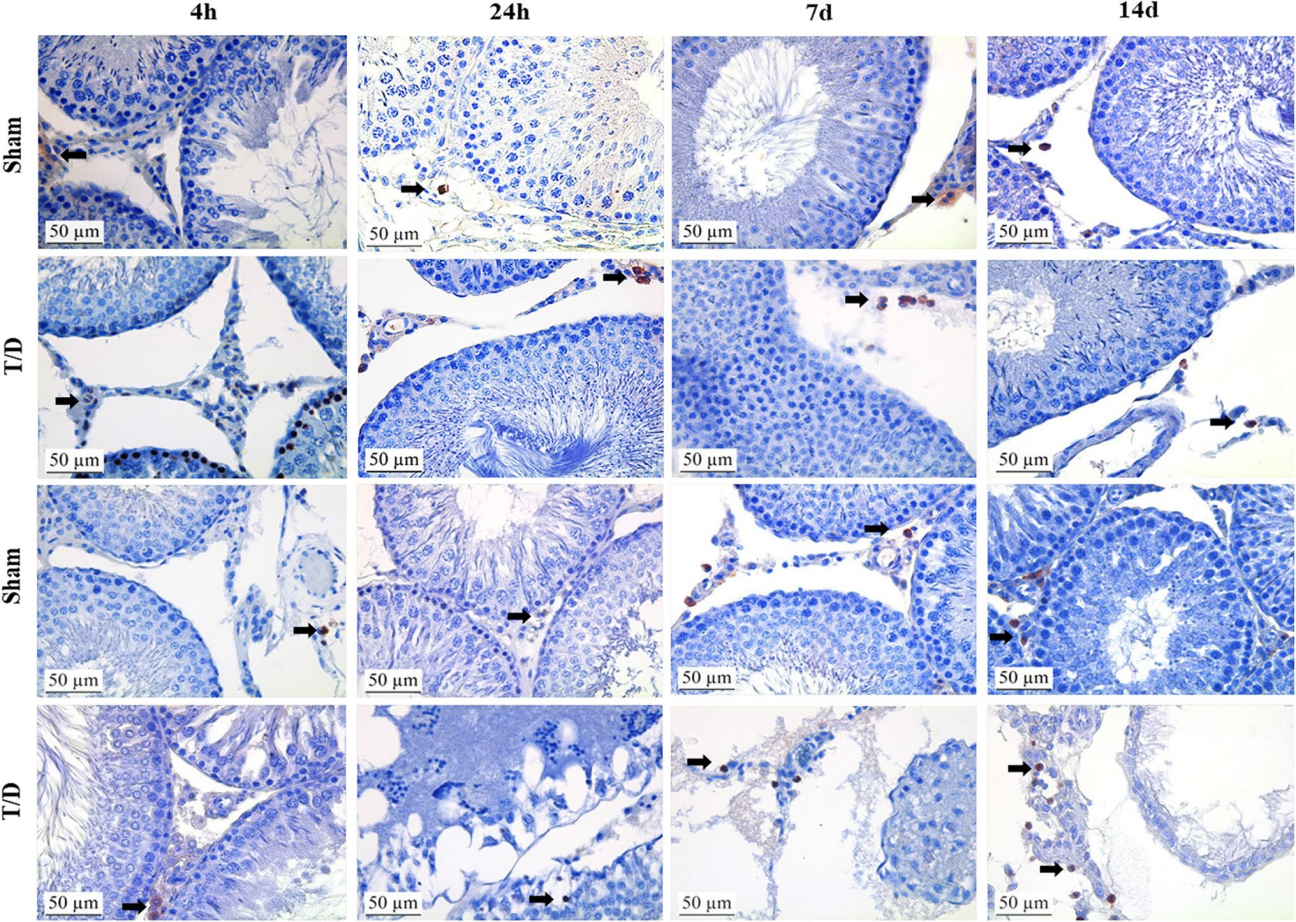
Immunohistochemical analysis of CD163 protein expression in testis tissue. Positive staining is indicated by a dark brown color (black arrows). All slides were counterstain with hematoxylin, scale bar: 50 μm,  × 400

An increase in CD163 immunoreactivity was observed in the contralateral testicular tissues in the T/D 7d group (*p* < 0.05), while the CD163 immunoreactivity was similar in the T/D 4 h, T/D 24 h and T/D 14d groups. A comparison of the T/D 4 h group with the T/D 14d group revealed an increase in CD68 immunoreactivity, reaching the highest score in the T/D 14d group (Fig. 3). The CD68 and CD163 immunoreactivity histoscores in testicular tissue are presented in Table 3.

**Table 3:** CD68 and CD163 Immunoreactivities

		Ipsilateral (Left)		Contralateral (Right)	
	N	CD 68	CD 163	CD 68	CD 163
Control	10	0.080 ± 0.024^ı^	0.170 ± 0.036	0.060 ± 0.026	0.190 ± 0.069
Sham 4h	10	0.060 ± 0.022^ı^	0.120 ± 0.024^ h^	0.050 ± 0.016^ı^	0.120 ± 0.038^ h^
Sham 24h	10	0.070 ± 0.021^ı^	0.160 ± 0.034^ h^	0.070 ± 0.02^ı^	0.130 ± 0.033^ h^
Sham 7d	10	0.060 ± 0.026^ı^	0.140 ± 0.034^ h^	0.080 ± 0.029^ı^	0.090 ± 0.027^ h^
Sham 14d	10	0.050 ± 0.016^ı^	0.090 ± 0.027^ h^	0.060 ± 0.022^ı^	0.110 ± 0.027^ h^
Torsion 4 h	10	0.190 ± 0.037	0.190 ± 0.037	0.180 ± 0.038	0.130 ± 0.033^ h^
Torsion 24h	10	0.110 ± 0.030	0.140 ± 0.026	0.220 ± 0.041	0.250 ± 0.045^ h^
Torsion 7d	10	0.160 ± 0.030	0.250 ± 0.026^e^	0.270 ± 0.053	0.620 ± 0.041^a^
Torsion 14d	10	0.260 ± 0.049	0.190 ± 0.031	0.320 ± 0.049^a^	0.270 ± 0.036^d^
*p* values		0.001	< 0.001	< 0.001	< 0.001

Values are presented as mean ± standard error. ^a^: Compared to the Control group; ^b^: Compared to the Sham 4 h group; ^c^: Compared to the Sham 24 h group; ^d^: Compared to the Sham 7d group; ^e^: Compared to the Sham 14d group; ^f^: Compared to the Torsion 4 h group; ^g^: Compared to the Torsion 24 h group; ^h^: Compared to the Torsion 7d group; ^ı^: Compared to the Torsion 14d group; *p* < 0.05

### Sperm Analysis

The statistical analysis of the sperm motility values of the experimental groups revealed, a significant decrease in motility in the T/D groups when compared to the Control group (*p* < 0.05).

A between-group comparison of sperm density and the proportion of abnormal sperm heads, abnormal tails and total abnormal sperm revealed no significant differences (*p* > 0.05) (Table 4).

**Table 4:** Sperm morphological analysis

	Motility (%)	Intensity (million/right cauda epididymis)	Abnormal sperm rate (%)		
			Head	Tail	Total
Control	80.00 ± 3.16	81.60 ± 9.32	4.00 ± 0.44	3.80 ± 0.66	9.20 ± 0.73
Sham 4h	74.00 ± 2.44	73.60 ± 7.13	3.20 ± 1.02	5.40 ± 1.07	10.00 ± 2.58
Sham 24h	72.00 ± 2.00	96.00 ± 5.13	3.80 ± 0.91	3.60 ± 0.67	7.40 ± 0.81
Sham 7d	76.00 ± 4.00	88.60 ± 9.94	3.20 ± 0.58	4.00 ± 0.83	7.40 ± 0.60
Sham 14d	68.00 ± 3.74	90.00 ± 7.07	3.40 ± 0.51	3.00 ± 0.77	6.40 ± 0.51
Torsion 4h	46.00 ± 5.09^a^	76.20 ± 7.44	4.20 ± 0.80	5.80 ± 0.86	10.00 ± 0.54
Torsion 24h	46.00 ± 5.09^a^	90.40 ± 4.62	5.20 ± 1.06	3.80 ± 0.66	11.20 ± 1.98
Torsion 7d	2.00±2.00^a.b.c.d.e.f.g^	64.80 ± 8.08	6.40 ± 0.92	5.20 ± 0.86	9.60 ± 1.47
Torsion 14d	2.00±2.00^a.b.c.d.e.f.g^	70.80 ± 9.47	5.00 ± 0.70	3.80 ± 0.66	9.40 ± 1.32
*p* values	< 0.001	0.144	0.221	0.217	0.115

Values are presented as mean ± standard error. ^a^: Compared with the Control group; ^b^: Compared with the Sham 4 h group; ^c^: Compared with the Sham 24 h group; ^d^: Compared with the Sham 7d group; ^e^: Compared with the

Sham 14d group; ^f^: Compared with the T/D 4 h group; ^g^: Compared with the T/D 24 h group; ^h^: Compared with the T/D 7d group; ^ı^: Compared with the T/D 14d group; *p* < 0.05

### Biochemical Analyses

The ELISA analyses revealed testosterone levels to be significantly decreased in both the Sham and T/D groups when compared to the Control group (*p* < 0.05), although the testosterone levels were similar within the T/D groups (*p* > 0.05). A comparison with the Control group revealed FSH levels to be decreased in the T/D 24 h, T/D 7d, and T/D 14d groups (*p* < 0.05). Furthermore, LH levels were noted to be affected in all groups, with notably lower LH levels recorded in the T/D groups when Compared with the Control group (*p* < 0.05). The changes in serum FSH, LH and testosterone levels due to testicular torsion are presented in Table 5.

**Table 5:** Effect of testis torsion on serum FSH, LH and testosterone levels

	N	FSH	LH	Testosterone
Control	5	8.283 ± 0.219	7.346 ± 0.167	483.900 ± 9.651
Sham 4 h	5	8.282 ± 0.391	5.997 ± 0.308	390.320 ± 22.485^a^
Sham 24 h	5	8.701 ± 0.577	6.147 ± 0.263	365.520 ± 5.646^a^
Sham 7d	5	7.888 ± 0.347	6.325 ± 0.399	354.760 ± 8.053^a^
Sham 14d	5	7.638 ± 0.651	6.323 ± 0.111	374.680 ± 4.135^a^
Torsion 4 h	5	7.089 ± 0.112	5.993 ± 0.131^a^	373.600 ± 3.062^a^
Torsion 24 h	5	6.575 ± 0.191^a,b,c^	5.746 ± 0.174^a^	367.640 ± 6.081^a^
Torsion 7d	5	6.491 ± 0.067^a,b,c^	5.666 ± 0.260^a^	370.640 ± 3.162^a^
Torsion 14d	5	6.602 ± 0.204^a,b,c^	5.416 ± 0.168^a^	377.480 ± 4.005^a^
*p* values		< 0.001	0.006	< 0.001

Values are presented as mean ± standard error. ^a^: Compared with the Control group; ^b^: Compared with the Sham 4 h group; ^c^: Compared with the Sham 24 h group; ^d^: Compared with the Sham 7d group; ^e^: Compared with the Sham 14 d group; ^f^: Compared with the T/D 4 h group; ^g^: Compared with the T/D 24 h group; ^h^: Compared with the T/D 7d group; ^ı^: Compared with the T/D 14d group; *p* < 0.05

## Discussion

In the present study, 0testicular injury after unilateral T/D was found to cause damage in the contralateral testis, to bring about changes in M1/M2 macrophage polarization and to increase M1. macrophages, especially in the contralateral testis. This suggests that not only ischemic injury but also immune system-mediated mechanisms play a role in T/D.

A previous experimental study reported that changes in the tissue started to appear from the first half hour after testicular torsion as a result of impaired blood flow in a 30 min 720° left testicular torsion model, and irregularities were observed in the germinal epithelium of the seminiferous tubule in the rat testicular tissue 1 h after reperfusion, with gradually increased degeneration after 24 h. After 48 h, bleeding and congestion occurred, and the spilling of immature cells into the seminiferous tubule lumen was observed. The study also reported the presence of multinucleated giant cells in the germinal epithelium 7 days later, and the severity and prevalence of histopathological findings in the testes tissue were at the highest level in this study [23]. In another study, after 5 h of ischemia, focal hemorrhage, congestion, interstitial edema, metaphase arrest and separations in the germinal epithelium were reported in the testicular tissue, and were more common in the ipsilateral testes than in the contralateral [24]. In the present study, minimal edema, congestion and separations in the germinal epithelium were observed in the contralateral testicular tissues of the T/D 4 h and T/D 24 h groups, and much more severe histopathological findings were noted in the ipsilateral testicles than in the contralateral testicles, concurring with previous literature. The presence of immature germ cells spilling into the tubule lumen on this side was also observed. In the T/D 7d and T/D 14d groups in particular, seminiferous tubule damage, multi-nucleated giant cell numbers and metaphase arrest in the germ cells were observed to be increased in the ipsilateral testes, aligning with the results of previous studies in literature. The necrotic tubules observed in the ipsilateral testes of the T/D 14d group support the progression of the pathological process on this side.

Decreases in the seminiferous tubule diameter and germinal epithelial thickness are associated with degeneration of the spermatogenic cell line. Shokoohi et al. (2018) reported a significant decrease in seminiferous tubule diameters and germinal epithelial thicknesses in the ipsilateral testes on day 14 after 4 h of testicular torsion [25]. In another study, seminiferous tubule diameters and germinal epithelium thicknesses were reported to show only minimal reductions in the ipsilateral testes 2 h after T/D [26]. The fact that these morphometric parameters, which started to decrease after T/D, showed a severe decrease on day 14 is consistent with the suggestion that the severity of ischemic testis damage is dependent on the duration and degree of torsion [27]. In the present study, significant decreases were noted in the seminiferous tubule diameters and germinal epithelium thicknesses of the ipsilateral testes, particularly in the T/D 14d group, supporting the findings of previous studies in literature. There was, however, no significant change in either parameter in the contralateral testes, which can be explained by the relatively weak histopathological changes in the ipsilateral testes.

Dejban et al. reported that the Cosentino scores increased while the Johnsen scores decreased in both ipsilateral and contralateral testis in their T/D groups in a comparison with their Control groups in a 7-day study [28], and they attributed the increased Cosentino score to compensatory hypertrophy in the contralateral testis tissue. In another study, a decrease in Johnsen scores was reported on both sides, especially in the ipsilateral testis, 24 h after 4 h of torsion [29]. In the present study, the Cosentino scores increased significantly in the T/D 7d and T/D 14d groups, especially in the ipsilateral testicles, and similarly, the Johnsen scores decreased gradually in both testicular torsion groups, supporting our histopathological findings.

In a long-term study evaluating bilateral testicular torsion, sperm parameters were reported to be impaired following torsion, with the most affected parameter being motility [30].

As the number of comprehensive studies evaluating the effects of torsion on sperm parameters is limited, the unchanged parameters in semen analyses performed in the early period after torsion cannot be considered an objective reflection of the effect of testicular torsion on fertility [5]. In the present study, a gradual decrease in sperm motility was noted in the torsion groups from 4 h to 14 days, while no significant difference was observed between the groups in terms of abnormal sperm rates and sperm density, which can be attributed to the limitation of sperm analysis performed mostly 14 days after T/D.

Cho et al. (2010) reported a decrease in plasma LH concentrations in both the sham and torsion groups in their study, and suggested that the reduction in this hormone could be associated with the effect of stress on the hypothalamus-pituitary–gonadal axis. The same study reported serum testosterone levels to be decreased as a result of the reduction of LH, while an evaluation of FSH levels – an indicator of spermatogenesis – revealed a minimal decrease in the torsion groups [31]. In the present study, decreases in serum FSH, LH and testosterone levels were detected in the torsion groups when compared with the control group, which supports the previous findings in literature.

Despite the long history of studies of the issue, the mechanism behind contralateral testis damage remains unclear. Various studies to date have suggested that contralateral testis damage may occur due to the effects of free oxygen radicals, through autoantibodies, and as a result of changes in blood flow or changes in enzyme levels in the contralateral testis after testis detorsion [32]. Although there are numerous experimental studies in the literature investigating unilateral testicular torsion/detorsion, there has been no study to date investigating whether an increase occurs in the M1 macrophages, which can cause high tissue damage in the interstitial area, to determine macrophage polarization and to explain contralateral testis damage. In the present study, it was determined that in the rat testis tissues of the Control and Sham groups, CD163-positive. macrophages, as M2 macrophage markers, were identified in greater numbers in the interstitial spaces when compared to the CD68-positive macrophages, as M1 macrophage markers. In the groups in which T/D was applied, however, changes were detected in the M1/M2 macrophage polarization dynamics, which is consistent with earlier studies reporting the possible polarization of macrophages into M1 cells via IFN-γ, or M2 cells via IL-4, and the potential of their phenotypes to change under various physiological and pathological conditions [15, 16, 33]. Macrophages operate through two separate pathways when carrying out their different functions, being the classic and alternative routes. Macrophages activated through the classic route and termed M1 trigger inflammation. M1 macrophages exhibit high phagocytosis and lead to tissue damage [34]. In the present study it is worthy of note that in the groups in which T/D was applied, the immunoreactivity of CD68 and CD163 in the contralateral testis tissue was greater than in the ipsilateral testis tissue. In the ipsilateral testis tissues, CD68 immunoreactivity increased significantly in the T/D 14d group when compared to the Control group, and increased from the T/D 4 h group to the T/D 14d group, reaching its highest value in the T/D 14d group. This suggests an increase in M1 macrophages, which can cause tissue damage in the contralateral testis tissue in unilateral testicular T/D. An evaluation of CD163 immunoreactivity revealed a significant increase in the T/D 7d group in both the ipsilateral and contralateral testis tissues that was more pronounced in the contralateral testis tissue. The increased CD163 immunoreactivity in both the ipsilateral and contralateral testis tissues on the 7th day of torsion may also be a protective mechanism, indicating an increase in tissue-repairing M2-type macrophages for the protection of both tissues. M2 macrophages can convert to M1 macrophages [34, 35], although in the present study it was determined that rather than transforming from one to the other in M1/M2 macrophage polarization, both CD68- and CD163-positive macrophages generally increased in number in the testis tissues. In the T/D 14d group in the present study, both ipsilateral and contralateral CD68-positive M1 macrophages increased in number when compared to the other days, although this increase on the 14th day may also be attributable to the phagocytosing mechanism behind the cleaning of the remnants of intense tissue and cell damage.

To conclude, the findings of the present study suggest that testicular damage occurs not only in the ipsilateral testis but also in the contralateral testis after unilateral testicular T/D. The most noteworthy finding of the present study is its determination that not only oxidative stress and free radical scavengers play a role in the pathogenesis of ischemia–reperfusion injury, but also changes in M1/M2 macrophage polarization in rat testicles. Further and more detailed studies are required to fully elucidate the role of the mechanisms associated with immune system cells.

## Data Availability

Not applicable.
